# Comparative Study of Transmission of 2940 nm Wavelength in Six Different Aesthetic Orthodontic Brackets

**DOI:** 10.3390/dj11030067

**Published:** 2023-03-01

**Authors:** Mohammad Khare Zamzam, Omar Hamadah, Toni Espana-Tost, Josep Arnabat-Dominguez

**Affiliations:** 1 Department of Odontostomatology, Faculty of Medicine, University of Barcelona, 08907 Barcelona, Spain; 2 Higher Institute of Laser Research and Applications, Damascus University, Damascus 222, Syria

**Keywords:** transmission, spectrophotometer, ceramic, aesthetic bracket, debonding, infrared, hard tissue laser, erbium laser, adhesive resin, thermal softening, thermal ablation

## Abstract

Background: Previous studies have confirmed the superiority of using erbium lasers (2940, 2780 nm) over other lasers in the debonding of ceramic brackets due to their safety and effectiveness. The most important factor in the debonding of aesthetic brackets is the transmission of the erbium laser through the aesthetic bracket to the adhesive resin. Objective: To identify the transmission of the 2940 nm wavelength through different types of aesthetic brackets. Materials and methods: A total of 60 aesthetic brackets were divided into six equal groups (**10** monocrystalline sapphire brackets—Radiance, AO; **10** monocrystalline sapphire brackets—Absolute, Star Dentech; **10** polycrystalline brackets—20/40, AO; **10** polycrystalline brackets—3M Unitek Gemini Clear Ceramic; **10** silicon brackets—Silkon Plus, AO; **10** composite brackets—Orthoflex, OrthoTech). The aesthetic brackets were mounted in a Fourier transform infrared spectrophotometer (FTIR IRPrestige-21, SHIMADZU) following the typical spectroscopy lab procedure for such samples. The transmission ratio for the 2940 nm wavelength was obtained using IRsolution software. The mean transmission values of the tested groups were compared using a one-way analysis of variance (ANOVA) test followed by a Bonferroni test (post-hoc test). Results: The highest transmission ratio was observed for the Radiance sapphire brackets (64.75%) and the lowest was observed for the 3M polycrystalline brackets (40.48%). The differences among the Aesthetic brackets were significant (*p* < 0.05). Conclusions: The thick polycrystalline and composite brackets have the lowest transmissibility, whereas the monocrystalline sapphire brackets have the highest transmissibility for the 2940 nm wavelength, meaning that there is a higher possibility of debonding them with a hard tissue laser through thermal ablation.

## 1. Introduction

The demand for more aesthetic orthodontic appliances has become higher, especially in adult patients, which has led to a significant increase in the demand for aesthetic ceramic and non-ceramic brackets for fixed orthodontic treatment [1].

The first aesthetic brackets were introduced in orthodontics by Newman in 1965, and ceramic brackets appeared in the 1980s [2]. There are two types of ceramic brackets, according to their structure: polycrystalline and monocrystalline sapphire with mechanical or chemical retention [2,3]. Polycrystalline ceramic brackets reflect the light, resulting in some degree of opacity. In contrast, monocrystalline brackets permit the passage of light, making these brackets basically clear [4]. Nowadays, there are zirconia brackets, which are considered ceramic brackets, but little information about them is available, and they need to be tested more [5].

Non-ceramic brackets are another type of aesthetic bracket, and this category includes polymer and plastic (silicon) brackets. These brackets were developed in response to reports of enamel damage during the debonding of ceramic brackets by mechanical methods, and because of excessive wear of the enamel surfaces of opposing teeth [6,7]. Such brackets are useful for short-duration orthodontic treatment as the applied force is minimal [8]. Unfortunately, these types of aesthetic brackets exhibit some disadvantages because of their low fracture toughness, low modulus of elasticity, slot distortion, deformation, discoloration, and inability to hold out the torquing forces generated by orthodontic rectangular wire [8,9]. Therefore, considerable research into reinforcement methods for these aesthetic brackets, including reinforcement by fibers or the use of metallic slots, has been undertaken, [9,10].

One of the challenges following the completion of orthodontic treatment is the debonding of aesthetic brackets, as this should be achieved without causing enamel damage or discomfort to the patient [11,12,13,14].

The debonding of composite and plastic brackets is accomplished using metal pliers with a special design, and the mechanical debracketing techniques include the slow application of a squeezing force to the bracket. The blades of these specially designed pliers work within the adhesive resin so that the debonding force is applied at the bracket–adhesive interface, and on both sides simultaneously [15,16].

In contrast, the debonding of ceramic brackets is more difficult due to the properties of these brackets, such as their hardness, low tensile strength, low fracture toughness, high bonding resistance, high modulus of elasticity, and low flexibility, which lead to complications during debonding. The debonding force therefore has little ability to remove these rigid brackets after orthodontic treatment is concluded [1].

Many ways to decrease the side effects associated with the debonding of ceramic brackets are available. These approaches include mechanical, ultrasonic, electrothermal, and laser debonding methods [3,17,18].

Three mechanical debonding techniques have been described: wrenching, delamination, and lift-off [19,20,21]. Though many manufacturers have introduced debracketing pliers with special designs for their trademark ceramic bracket, and they allege that their ceramic brackets can be removed as easily and safely as metal brackets if the specialist orthodontist follows their instructions [22,23,24], mechanical debracketing still leads to bracket fracture, and the force applied causes discomfort to the patient [17,25]. Ceramic bracket fragments may detach or stay attached to the tooth surface, so the removal of these fragments using low- or high-speed handpieces under water cooling is necessary [26].

The ultrasonic debonding technique exhibits a decreased probability of bracket breakage and a decreased probability of enamel damage. In addition, the residual adhesive resin on the enamel surface after debonding can be removed with the same ultrasonic tip. The debonding time is still the longest, however, when compared with electrothermal or mechanical debonding techniques. Moreover, the contact between the ultrasonic tip and ceramic bracket has been reported to cause wear of the ultrasonic tip, which is expensive [17,27].

It has been reported that electro-thermal debracketing can reduce the incidence of ceramic bracket breakage because the heat-induced tip can induce bond weakening by softening the adhesive resin. As a result, only a low-level force is needed to break the bond [28]. Unfortunately, a possibility of pulp damage has been reported [29].

The implementation of lasers in the orthodontics field has increased in recent years with an increased focus on the debonding of ceramic brackets using different laser sources such as neodymium-doped yttrium aluminum garnet (Nd:YAG 1064 nm) [30], CO_2_ (10,600 nm) [31], TM:YAP [32], ytterbium-doped fiber [33], and assorted diode lasers [34]. Due to their safety and effectiveness, previous studies have confirmed the superiority of using erbium lasers (Er:YAG 2940 nm, ErCr: YSGG 2780 nm) and, to a lesser degree, CO_2_ lasers (10,600 nm) over other lasers for the debonding of ceramic brackets [2,12,35].

The debracketing of ceramic brackets using erbium lasers has become an approved technique in the scope of orthodontics as the problems associated with the debonding of ceramic brackets using conventional methods are avoided when this laser is used [36]. These problems may include pain experienced by the patient during the removal of the ceramic bracket and enamel cracking and fracturing [33,37]. Moreover, the use of a laser reduces the time and effort needed for the debonding of brackets [38].

Debonding with Er: YAG lasers (2940 nm) is still controversial among researchers because, according to some of them, the thermomechanical ablation that occurs in the superficial layer of the adhesive resin after the irradiation of the ceramic bracket with an erbium laser could lead to bond weakening [39]. Per contra, some researchers have found that the dominant effect is thermal softening, which weakens the adhesion strength between the resin and the ceramic bracket base [36,40].

The laser debonding procedure is highly affected by the type of ceramic bracket as well as by the type of laser. It has been reported that monocrystalline and polycrystalline ceramic brackets have shown different reactions to laser light at different wavelengths due to their different optic characteristics [41], as monocrystalline ceramic brackets are more suitable for laser debonding than polycrystalline ceramic brackets [3].

The most important factor in aesthetic bracket debonding is how much the laser transmits through the bracket to reach the adhesive resin [35,42,43].

Through the introduction of spectroscopy technology in dentistry, we can obtain a lot of information concerning dental structures as well as dental material, including aesthetic brackets [44,45]. One of these spectroscopy technologies is Fourier transform infrared (FTIR) spectroscopy, which is considered a non-destructive and label-free vibrational spectroscopic technique and is widely used in research [44]. Dental material can be tested by FTIR in transmission mode [46].

Due to the absence of studies that investigate the transmission of infrared light through all types of aesthetic brackets, our study aimed to spectroscopically investigate the transmission of the 2940 nm wavelength through different types of aesthetic brackets which are available for clinical use.

The null hypothesis is that the transmission values of the 2940 nm wavelength through different aesthetic brackets are the same.

## 2. Materials and Methods

**Study design**: 

It is an in vitro study. 

**Sample**: 

The sample consisted of 60 different aesthetic brackets for upper premolars MBT 0.022, divided into six equal groups. Two groups contain monocrystalline sapphire brackets from different companies, two groups contain polycrystalline brackets from different companies, one group contains silicon brackets, and one group contains composite brackets, as is shown in Table 1.

**Fourier Transform Infrared Spectrophotometer:**

**Figure 1:**
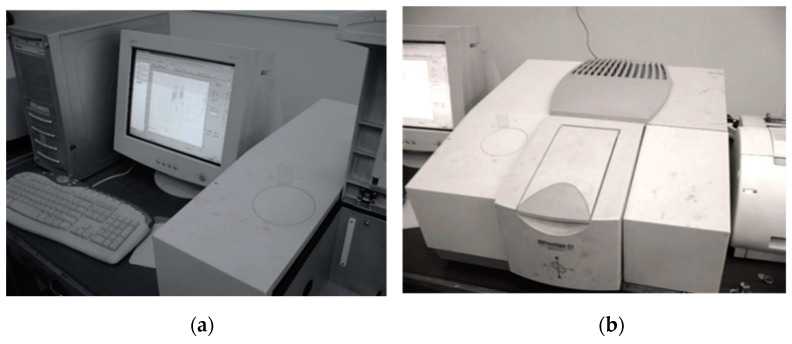
IRPrestige-21, infrared spectrophotometer: (**a**) desktop computer; (**b**) interferometer.

This device measures an infrared spectrum (wave numbers) obtained from the Fourier-transform of an interferogram. The device we used is an IRPrestige-21 from SHIMADZU^®^ (Tokyo, Japan 2002) (Figure 1).

The IRPrestige-21 uses a bright ceramic light source (wavenumber measurement range 7500–400 cm^−1^) (1.33–25 µm), a high-sensitivity DLATGS detector, and high-throughput optical elements (Table 2). The IRPrestige-21 depends on a Michelson interferometer, which is considered one of the most important parts of the FTIR spectrophotometer. After passing the aperture, the emitted light is turned into a parallel beam by the collimator mirror and enters the beam splitter. As the single beam is split into two beams, one is reflected in the fixed mirror and the other is transmitted to the moving mirror. Both mirrors reflect their beams back to the beam splitter, and part of each returning beam is reflected and transmitted. The reflected light from the moving mirror and the transmitted light from the fixed mirror recombine and interfere before traveling towards the collecting mirror. This recombination can be either destructive or constructive interference [47].

**Transmission Spectrum Study of Aesthetic Orthodontic Brackets**: 

This study was conducted by a specialist engineer at the Higher Institute for Laser Research and Applications (HILRA), Damascus University, Syria. All of the required instructions were followed in the HILRA spectroscopy lab.

**Figure 2:**
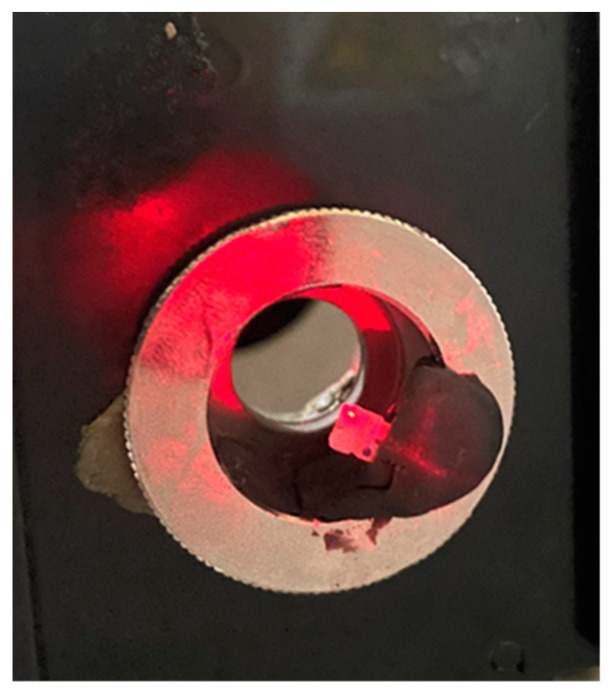
Positioning of the aesthetic bracket with the laser aimed at the occlusal distal wing.

Each sample (aesthetic bracket) was prepared and mounted in the spectrophotometer following the typical lab procedures for such samples. Since the aiming laser (He–Ne class II laser) matched the occlusal distal wing of the aesthetic bracket, the wavelengths would pass through the thickest part of the aesthetic bracket (Figure 2).

The transmission study was carried out by following all the steps proposed by the manufacturer, taking air as the reference for transmittance (100% transmission). “IRsolution” software was used and the measurements appeared as a chart and readings (wavenumber or wavelength to transmittance ratio), which could easily be saved as a BMP file and a text file ready for statistical study.

### Statistical Analysis

Descriptive statistics were generated for the transmission values as a percentage, including mean, standard deviation, and maximum and minimum values. The mean transmission values were compared between the six test groups using a one-way analysis of variance (ANOVA) test followed by a Bonferroni test (post-hoc test). The normal distribution of the data and the test of homogeneity of variances were confirmed using Kolmogorov–Smirnov and Levene’s tests. The significance was determined at a probability value of *p* < 0.05 for all statistical tests in this study. The statistical analysis was performed using IBM^®^ SPSS^®^ statistics version 17.

Regarding the calculation of the required sample size, we used G*Power version 3.1.2. The sampling power was 95% for the current study, and the calculated sample size according to the G*Power software was 60 brackets divided into 6 equal groups.

## 3. Results 

### Effect of Type of Bracket on Transmission Ratio Values at 2940 nm Wavelength

A one-way ANOVA test (Table 3) was applied to determine whether there were significant differences in the transmission ratio values between the different bracket groups (Radiance, Absolute, 20/40, 3M, Silkon Plus, and Composite Ortho Flex), as is shown below:

- One-Way ANOVA test results:

All of the *p*-values were much lower than 0.05, so, at a confidence level of 95%, there were significant differences in the transmission ratio values between at least two of the subgroups. A Bonferroni post-hoc test (Table 4) was therefore applied to determine whether there were pair-wise significant differences in transmission ratio values between different bracket groups, as is shown below.

**Figure 3:**
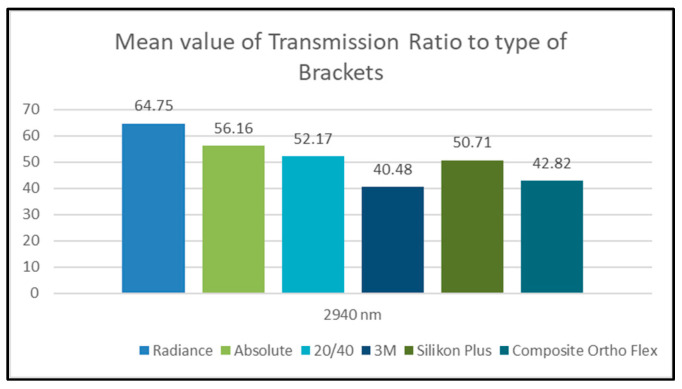
Mean of transmission ratio values according to type of bracket at 2940 nm wavelength.

- Bonferroni post-hoc test results:

All of the *p*-values were much lower than 0.05, so, at a confidence level of 95%, there were significant pair-wise differences in the transmission ratio values between all of the bracket groups, regardless of the wavelength group in the sample. According to the algebraic sign of mean differences, we found that:

- The transmission ratio values of the Radiance group were greater than those of all the other groups.
- The transmission ratio values of the Absolute group were greater than those of four other groups (20/40, 3M, Silkon Plus, and Composite Ortho Flex).
- The transmission ratio values of the 20/40 group were greater than those of three other groups (3M, Silkon Plus, and Composite Ortho Flex).
- The transmission ratio values of the Silkon Plus group were greater than those of both the 3M and Composite Ortho Flex groups.
- The transmission ratio values of the Composite Ortho Flex group were greater than those of the 3M group (Figure 3).

## 4. Discussion

The main factor in orthodontic bracket debonding is the weakening of the bonding strength of the adhesive resin connecting the bracket to the enamel surface [48,49]. Such modulation can be achieved by applying a laser which partially breaks through the ceramic bracket towards the adhesive material and therefore manipulates the strength of its bond to the tooth surface [50]. As the Er:YAG laser (2940 nm) is absorbed well by water, the laser energy turns into heat within the bonding resin, resulting vapor and the debonding of the ceramic bracket from the tooth [40,51]. However, the laser’s power to penetrate of is affected by the bracket material [35].

It has been reported that the material from which the ceramic bracket is made, its morphological factors, and its composition can influence the penetration of direct light significantly. Polycrystalline ceramic brackets have been found to block the direct transmittance of light, whereas monocrystalline brackets have been observed to permit the passage of more light [41,43,45].

However, no studies have been carried out on the transmission of the 2940 nm wavelength through different types of aesthetic orthodontic brackets. Our study has been conducted in order to test the transmission of this specific wavelength, which is used for debonding of aesthetic brackets with lasers.

Regarding the effect of the type of bracket on transmission values, the obtained results of our study totally refute the null hypothesis and support the alternative hypothesis of the existence of significant difference in transmission values between different types of aesthetic brackets at the 2940 nm wavelength. The 2940 nm wavelength had the highest transmission value through the Radiance brackets (64.75%), followed by the Absolute brackets (56.16%), 20/40 brackets (52.17%), Silkon Plus brackets (50.71%), Composite Ortho Flex brackets (42.82%), and, finally, the 3M brackets, which gave the lowest transmission value (40.48%).

Briefly, the highest transmission values for the studied wavelength were observed in the sapphire brackets (Radiance and Absolute), whereas the lowest values were observed in the 3M polycrystalline brackets. The Silicon and 20/40 brackets gave transmission values in the middle.

Our results indicate that two-thirds of the initial intensity penetrate the Radiance sapphire brackets, and about half of the initial intensity penetrates the Absolute, 20/40, and Silkon Plus brackets, while less than half of the initial intensity penetrates the Composite Ortho Flex and 3M brackets.

By having a close look at the detailed results, we found that the transmission of the tested wavelength (2940 nm) differs according to the material structure of the bracket. For example, the transmission within the monocrystalline sapphire brackets was higher than that that within the polycrystalline brackets and the other aesthetic brackets considered in this study. This agrees with results obtained in other studies, i.e., that the spectral transmissibility within polycrystalline brackets is low compared with the transmissibility within monocrystalline (sapphire) brackets [41,45,52]. This means that, in order to achieve ablative debonding, greater density of energy and pulse power are required from the laser [35,48]. The greater the transmission, the less energy density is required, and vice versa.

Another important factor which affects the transmission value is the thickness of the bracket. We found that there is a significant difference between the 20/40 and 3M brackets, both of which are polycrystalline, as the transmission values of the 20/40 bracket were higher than those of the 3M bracket. However, when we measured the thickness of both brackets, we found that the thickness of the 20/40 bracket at the wing was less than that of the 3M bracket by 0.5 mm (1.8 mm for the 20/40 bracket, 2.3 mm for the 3M bracket). This result is in accordance with those of a study by Sari et al., which concluded that the laser power transmitted through the ceramic material decreases with the increasing thickness of the irradiated sample [53].

The last factor which affects the transmission values is the brand of the aesthetic bracket, i.e., how the company manufactures the brackets and the purity of the manufactured material [45]. A significant difference between Radiance and Absolute brackets has been noticed, as the transmission values of the Radiance brackets were higher than those of the Absolute brackets, though both are made of monocrystalline sapphire with the same thickness at the bracket’s wing. This observation is in accordance with that of a study by Mohamed et al. (2016), which found a significant difference in light transmittance between two brands of monocrystalline sapphire brackets [45].

When reviewing related studies, we found only a few studies that investigated the transmission of Er: YAG 2940 nm through ceramic brackets using a laser power meter and not a spectrophotometer. A comparison between our study and these studies will not therefore be credible or accurate.

Mundethu et al. (2013) found that the transmission of Er: YAG 2940 nm through a Damon Clear bracket (polycrystalline ceramic) is 85%, i.e., 510 mJ of the 600 mJ of the incident laser will interact with the bonding adhesive resin [43]. This transmission value is higher than the corresponding values for polycrystalline brackets obtained in our study (which were 52.17% for the 20/40 bracket and 40.48% for the 3M bracket), and we attribute this difference to the different measuring methods, and possibly to the location of the transmission measurement. In their study, the measurement was recorded at the slot of the bracket, whereas in our study, it was recorded at the wing of the bracket because the thickness is an important factor in transmission (more thickness, less transmission).

A study by Sari et al. (2014) compared the transmission of an Er: YAG 2940 nm laser through different ceramic restorations, and it found that 0.5 mm thick lithium disilicate-reinforced ceramic gave the highest transmission ratio (88%) and 1 mm thick feldspathic ceramic gave the lowest (44%) [53]. We cannot directly compare our results with the results of this study because of the differences in ceramic bracket structure and ceramic restoration. However, we agree with the authors about the importance of the thickness and type of ceramic in adjusting the laser irradiation parameters during the laser debonding of ceramic restorations and ceramic brackets.

Mesaros et al. (2022), in their systematic review of orthodontic bracket removal using laser technology, mentioned that the spectral transmissibility within polycrystalline brackets is low compared with the transmissibility within monocrystalline (sapphire) brackets [35]. This agrees with our results for the tested wavelength (2940 nm) within monocrystalline sapphire and polycrystalline brackets. This means that, in order to achieve ablative debonding, the energy density and pulse power of the laser should be greater.

Finally, we should mention that our study has some limitations relating to the infrared spectrophotometer we used. The device available in the lab was a standard one with basic accessories and was not supplied with equipment for reflection. We therefore could not measure the reflected light from the sample. Moreover, any obtained absorption values would not have been accurate because reflection events within the samples would have been ignored and transmission values would have been depended upon entirely. We therefore skipped the absorption values in our study.

A very important thing that should be pointed out is that while the transmission ratio values are a crucial and important factor in the debonding of aesthetic brackets, they are not the only factor. The absorption values are also important because the intensity absorbed by the aesthetic bracket affects the resin indirectly, potentially resulting in the thermal softening of the adhesive resin. Thus, the investigation of the absorption, reflection, and scattering within the aesthetic brackets in conjunction with the transmission spectrum is still necessary to obtain an overview of optical phenomena when using a hard tissue laser for the debonding of aesthetic brackets.

## 5. Conclusions 

Within the limits of our study, we may conclude the following:

1. Among the studied aesthetic brackets, the monocrystalline sapphire brackets have the highest transmissibility for the 2940 nm wavelength.
2. The thick polycrystalline and composite brackets have the lowest transmissibility for the 2940 nm wavelength.
3. The high transmission values of the tested wavelength within the monocrystalline sapphire brackets indicate that less pulse power and energy are required from the laser in order to achieve ablative debonding.
4. After testing all the other related factors, the good transmission values of the silicon brackets lead us to consider using a 2940 nm wavelength laser for debonding.
5. The relatively high transmission values of the monocrystalline sapphire brackets increase the possibility that they can be debonded using a hard tissue laser with a thermal ablation mechanism.

## Figures and Tables

**Table 1:** Sample description.

Sample				
Group N	Aesthetic Bracket	Trade Name	Company	Sample Size
1	Monocrystalline Sapphire	Radiance	American Orthodontics, Sheboygan, WI, USA	10
2	Monocrystalline Sapphire	Absolute	Star Dentech, Seoul, South Korea	10
3	Polycrystalline	20/40	American Orthodontics, USA	10
4	Polycrystalline	Unitek Gemini Clear	3M Unitek, Irwindale, CA, USA	10
5	Plastic (Silicon)	Silkon Plus	American Orthodontics, USA	10
6	Composite	Orthoflex	Ortho Technology, Tampa, FL, USA	10

**Table 2:** IRPrestige-21 specifications and parameters.

Item	Description
Interferometer	Michelson interferometer (incident angle is 30 °C)
Optical system	Single beam
Beam splitter	Ge coated on KBr
Light source	Cooling-type ceramic
Detector	DLATGS detector with temperature control
Wavenumber range	7500–400 cm^−1^ (±0.125 cm^−1^ accuracy)
Resolution	0.5 cm^−1^, 1 cm^−1^, 2 cm^−1^, 4 cm^−1^, 8 cm^−1^, 16 cm^−1^ (Mid/Far IR); 2 cm^−1^, 4 cm^−1^, 8 cm^−1^, 16 cm^−1^ (NIR)
S/N ratio	40,000:1
Mirror speed	2.8 mm/s, 5 mm/s, 9 mm/s, scanning at 4 cm^−1^ takes 2–3 s
Data sampling	He–Ne laser
Gain control	Automatic or manual (×1 to ×128)
Sample compartment	200 (w) × 230 (L) × 170 (H) mm, center focus
Dimensions	620 (w) × 680 (L) × 290 (H) mm
Weight	54 kg

**Table 3:** Results of the one-way ANOVA test to determine whether there were significant differences in the transmission ratio values between the different bracket groups at a 2940 nm wavelength.

Studied Variable = Transmission Ratio									
Wavelength	Type of Bracket	N	Mean	Std. Deviation	Minimum	Maximum	F Value	*p*-Value	Significant Diff.?
2940 nm	Radiance	10	64.75	0.40	64.05	65.27	5562.186	0.000	YES
	Absolute	10	56.16	0.55	55.54	56.72			
	20/40	10	52.17	0.14	51.99	52.34			
	3M	10	40.48	0.31	39.97	40.82			
	Silkon Plus	10	50.71	0.25	50.40	51.04			
	Composite Ortho Flex	10	42.82	0.46	42.21	43.51			

**Table 4:** Results of the Bonferroni post-hoc test to determine whether there were pair-wise significant differences in transmission ratio values between the different bracket groups according to wavelength.

Studied Variable = Transmission Ratio						
Wavelength	Type of Bracket (I)	Type of Bracket (J)	Mean Difference (I–J)	Std. Error	*p*-Value	Significant Diff.?
2940 nm	Radiance	Absolute	8.59	0.17	0.000	YES
		20/40	12.58	0.17	0.000	YES
		3M	24.26	0.17	0.000	YES
		Silkon Plus	14.04	0.17	0.000	YES
		Composite Ortho Flex	21.93	0.17	0.000	YES
	Absolute	20/40	3.99	0.17	0.000	YES
		3M	15.68	0.17	0.000	YES
		Silkon Plus	5.45	0.17	0.000	YES
		Composite Ortho Flex	13.34	0.17	0.000	YES
	20/40	3M	11.68	0.17	0.000	YES
		Silkon Plus	1.46	0.17	0.000	YES
		Composite Ortho Flex	9.35	0.17	0.000	YES
	3M	Silkon Plus	−10.23	0.17	0.000	YES
		Composite Ortho Flex	−2.33	0.17	0.000	YES
	Silkon Plus	Composite Ortho Flex	7.89	0.17	0.000	YES

## Data Availability

Not applicable.
